# The Potential of Multi-Screening Methods and Omics Technologies to Detect Both Regulated and Emerging Mycotoxins in Different Matrices

**DOI:** 10.3390/foods13111746

**Published:** 2024-06-02

**Authors:** Marco Lapris, Michela Errico, Gabriele Rocchetti, Antonio Gallo

**Affiliations:** Department of Animal Science, Food and Nutrition, Università Cattolica del Sacro Cuore, Via Emilia Parmense 84, 29122 Piacenza, Italy; marco.lapris@unicatt.it (M.L.); michela.errico@unicatt.it (M.E.); antonio.gallo@unicatt.it (A.G.)

**Keywords:** mycotoxins and masked mycotoxins, food/feed safety, mass spectrometry, multiscreening

## Abstract

Mycotoxins are well-known secondary metabolites produced by several fungi that grow and occur in different crops during both pre-harvest and post-harvest conditions. The contamination and occurrence of mycotoxins currently represent some of the major issues in the entire agri-food system. The quantification of mycotoxins in different feeds and foodstuffs is extremely difficult because of the low concentration ranges; therefore, both sample collection and preparation are essential to providing accurate detection and reliable quantification. Currently, several analytical methods are available for the detection of mycotoxins in both feed and food products, and liquid chromatography coupled with high-resolution mass spectrometry (LC-HRMS) represents the most reliable instrumental approach. In particular, the fast development of high-throughput methods has made it possible to screen and analyze, in the same analytical run and with high accuracy, multiple mycotoxins, such as those regulated, masked, or modified, and emerging ones. Therefore, the aim of this review is to provide an overview of the state of the art of mycotoxins occurrence, health-related concerns, and analyses, discussing the need to perform multi-screening approaches combined with omics technologies to simultaneously analyze several mycotoxins in different feed and food matrices. This approach is expected to provide more comprehensive information about the profile and distribution of emerging mycotoxins, thus enhancing the understanding of their co-occurrence and impact on the entire production chain.

## 1. Introduction

Mycotoxins are secondary metabolites produced by filamentous fungi belonging to different genera, including (among others) *Aspergillus*, *Penicillium*, *Fusarium*, and *Alternaria* [1,2,3,4]. These compounds have been the focus of research in several countries and are considered one of the major risk factors in agricultural products [5]. Among the main environmental factors contributing to the development of fungi-producing mycotoxins, it is possible to list humidity and temperature [6,7]. Tropical and subtropical climates represent optimal conditions for fungi growth; however, climate change is starting to dramatically affect continental climates, thus representing a great challenge for mitigation activities [8]. Mycotoxin contamination is one of the biggest threats in the agri-food chain, able, on the one side, to impact the global economy, and, on the other side, to force farmers to destroy infested crops [9]. Additionally, it can reduce animal performance and profitability, causing several health damage to animals, with corresponding economic losses [10,11]. Also, the simultaneous occurrence of mycotoxins in both feed and foodstuffs could potentially determine some synergistic effects in terms of toxicity to animals and humans [12,13].

**Figure 1:**
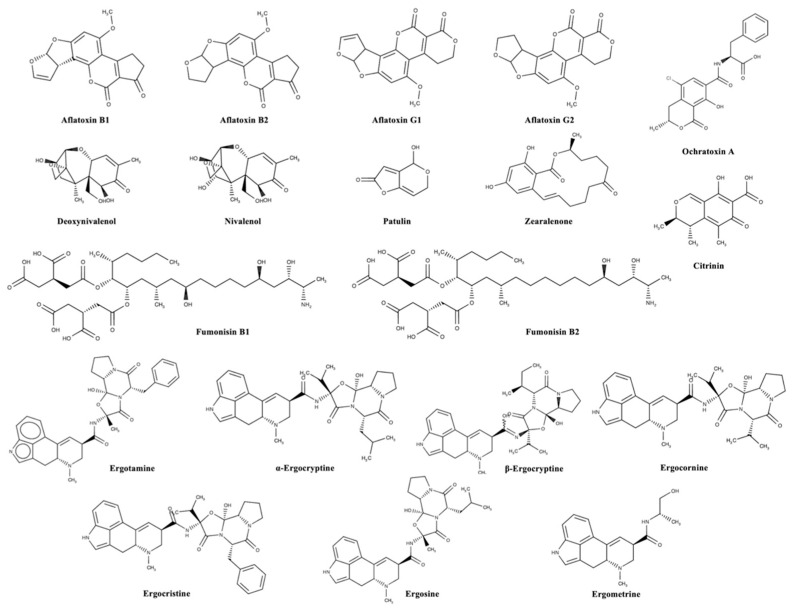
Chemical structures of the most important regulated mycotoxins in the agro-food chain.

**Figure 2:**
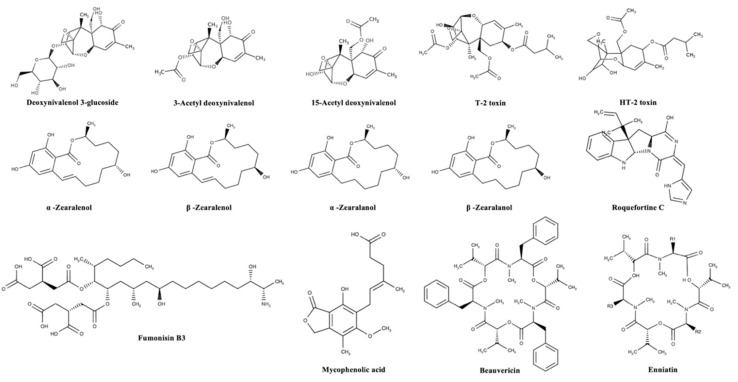
Chemical structures of the most important emerging mycotoxins in the agro-food chain.

Mycotoxins can be categorized according to substantial structural variation rather than their different origins; this huge diversity results in marked differences in their physical, chemical, and biological properties [14]. The chemical structures of the most important regulated and emerging mycotoxins are reported in Figure 1 and Figure 2, respectively.

The most relevant groups of mycotoxins for health concerns are represented by aflatoxins (AFs), fumonisins (FUMs), trichothecenes (TCs), zearalenone (ZEN), and ochratoxins (OTs) [15].

AFs have been defined as potent carcinogenic compounds by IARC [16]. These toxins are able to damage different organs, causing, among other things, hepatotoxicity, immunosuppression, and the severe alteration of coagulation processes [17,18]. In this regard, AFB1 can be listed as the most potent and harmful aflatoxin, mainly produced by *A. flavus* and *A. parasiticus* fungi [19,20]. The damaging effects triggered by AFs occur in both humans and animals. AFB1 exerts dangerous effects mainly on the liver, besides causing great challenges in terms of altered immune responses and gut health. On the other hand, FUMs are produced by *F. verticilliodes* and *F. proliferatum* and are mainly studied for their nephrotoxic and hepatotoxic activities [21].

Additionally, group A T-2 and HT-2 toxins can be categorized within the trichothecenes group, with the latter being characterized by more than 200 known compounds. T-2 and HT-2 toxins are produced by many species of fungi (such as *Fusarium*, *Trichoderma*, *Cephalosporium*, and others) and can negatively affect, among other things, heart muscle functionality and the immune system [22,23]. On the other hand, among the group B trichothecenes, it is possible to list DON. *F. graminearum* represents the main producer of this toxin, previously studied for its ability to inhibit protein synthesis; furthermore, other studies evaluated its high toxicity at the intestinal level and its ability to cause immune deficiency [24].

As far as ZEN is concerned, this toxin is mainly produced by *F. graminearum*. However, other species produce this toxin in lower quantities, such as (among others) *F. culmorum and F. verticillioides.* This toxin has been widely studied for its estrogenic properties, inducing reproductive disorders and immunotoxicity. This has been observed, especially in swine and rabbits [25,26]. Going into detail, ZEN promotes the release of different hormones, such as prolactin and luteinizing hormones, interfering with the normal estrus cycle, ovulation, and embryo implantation [27].

Finally, *Aspergillus* and *Penicillium* strains are reported to produce ochratoxins. The complete and accurate mechanisms of action of these toxins seem to be based on structural similarity with phenylalanine, determining an antagonist effect on the same biological targets. Overall, the most observed imbalance conditions are related to calcium homeostasis and affect protein synthesis [28]. OTA (belonging to the ochratoxins group) is a toxin first isolated from *A. ochraceus*. The most dangerous effects of this toxin are correlated with its nephrotoxic, immunotoxic, and genotoxic activities [29].

Starting from these background conditions, in this comprehensive review, we briefly describe the impact of mycotoxins on animal and human health and then we critically discuss the main findings on the available multi-screening methods to profile regulated and emerging mycotoxins in food, feed, and biological fluids. For the aim of this comprehensive review on mycotoxins, a search in the database Scopus was first carried out in order to identify related published reviews using the primary keywords “mycotoxins and masked mycotoxins”, “mass spectrometry”, and “multi-screening”. Afterwards, primary studies were searched in this database for studies with different combinations of the primary keywords to cover food, feed, and biological fluids in order to provide a comprehensive review of the most recent literature in this area. Additional searches in the Google Scholar engine were also carried out to enrich the references. Therefore, a total of 100 papers were finally considered in the context of this review.

## 2. Impact of Mycotoxins on Animal and Human Health

When talking about clinical and subclinical mycotoxicosis in animals, major mycotoxin exposure is usually related to the ingestion of contaminated feed [30]. In this regard, AFB1, DON, Fumonisins B (FBs), and ZEN are commonly found in grains and animal feeds. These toxins are widespread in agricultural products and animal feed and usually co-occur in the product, thus creating a major challenge in terms of toxicity [31]. Mycotoxin exposure determines different animal responses (depending on species), with sheep being described as relatively resistant when compared to poultry, piglets, and cattle [32,33]. As a general consideration, young poultry, rabbits, and swine are recognized as more susceptible to AFs [33], which are usually associated with anorexia, hemorrhages, edema, and jaundice. Swine are described as the most susceptible species to ZEN exposure, especially when considering young specimens and sows, while cattle and sheep are the most resistant to estrogenic side effects [34]. Equine and swine are highly susceptible to FUMs, with jaundice, edema, and severe dyspnea representing some of the most prominent and toxic effects. Ochratoxin exposure was previously reported to induce nephrotoxicity in laboratory animals, recording a maximum LD50 of 30.3 mg/kg bw, depending on the species [30]. As far as the group of ochratoxins is concerned, swine and poultry are extremely sensitive. Interestingly, swine may develop an exclusive condition known as porcine nephropathy [35]; in this regard, it is worth mentioning the Balkan endemic nephropathy, which has high relevance for Serbia, Bulgaria, Romania, Croatia, and Bosnia [36]. On the other hand, the main effect on poultry is related to an alteration in the intestinal microbiota, affecting meat pigmentation and eggshell fragility [30,37]. Several studies have reported that poultry, swine, and rabbits are the most susceptible species to trichothecene mycotoxins [30,38,39]. Farming animals are reported to suffer from vomiting, refusing feed, and changing feeding behavior. Finally, poultry, swine, and rabbits represent the most susceptible species to DON, with the latter being associated with oral ulcers and melena [40].

As comprehensively reviewed by Franchino et al. [30], acute mycotoxicosis on animal farms is rare because of the introduction of severe and improved animal feed hygiene standards. On the other hand, an opposite trend is followed by subclinical mycotoxicosis because of the low amount of mycotoxins in the feeding system, causing detrimental economic losses. Furthermore, the immunosuppressive effects of these fungal metabolites can potentially expose animals to other pathologies, thus reducing their ability to face less aggressive on-farm conditions. Mycotoxins can decrease animal resistance to environmental and microbial stressors, reducing their resilience and making them more susceptible to diseases [30]. Additionally, in the meat production area, mycotoxins can significantly compromise the overall quality of final products. Finally, all known mycotoxins are characterized by different levels of absorption, metabolism, and kinetics. Therefore, legislation connected with the mycotoxin contamination of animal feeds must consider all these parameters to properly identify and recommend limits for each mycotoxin (Table 1) [41,42,43].

Recently, Franchino and co-authors [30] showed some very interesting findings of a five-year monitoring plan dealing with the evaluation of AFB1, ZEN, DON, OTA, FUMs, and T-2/HT-2 toxins in 722 samples of animal feed. The authors revealed that fourteen samples were characterized by mycotoxin concentrations higher than related maximum residue limits (MRLs). In particular, non-compliant samples were associated with DON, AFB1, and ZEN, respectively, with maize being the most frequent raw material in these samples [30]. Also, the authors highlighted a potential correlation between mycotoxin type and structure and animal susceptibility.

According to both the European Food Safety Authority (EFSA) and the International Agency for Research on Cancer (IARC), some of these mycotoxins are classified as potentially dangerous for human health [44], with most of them causing toxic responses at low concentrations. As recently reported by Sdogati et al. [45], approximately 300 mycotoxins have been identified and characterized so far, but a very low number of them have relevance for both animal and human health. Mycotoxins can promote the generation of reactive oxygen species (ROS), thus inducing oxidative stress and the oxidation of DNA, proteins, and lipids [46]. For example, aflatoxins produced by *A. flavus* and *A. parasiticus* (such as AFB1, AFB2, AFG1, and AFG2) are bioactivated by cytochrome metabolism into exo-aflatoxin B1-8,9-epoxide (a highly reactive intermediate), leading to DNA damage during replication [47]. Also, aflatoxins can dramatically affect the liver and its overall functionality [48].

The consumption of mycotoxin-contaminated foods can lead to severe outbreaks [49]. Human exposure due to the transfer of mycotoxins from contaminated crops to the final food product represents one of the main exposure routes. Also, another possible route is related to the ability of animal metabolism to process and modify mycotoxins [45]. One of the most studied examples of the carry-over phenomenon is represented by the occurrence of AFM1, the hydroxylated metabolite of AFB1, in dairy products (i.e., milk and cheese). AFM1 presence in dairy products is a huge and severe health concern for humans; accordingly, this metabolite is characterized by high toxicity and carcinogenic properties. That is why the IARC classified AFB1 and AFM1 as human carcinogens belonging to Group 1 and Group 2B, respectively, because of their ability to form DNA adducts [50,51]. Animals consuming a diet potentially contaminated with mycotoxins are able to biochemically transform or process these toxins, distributing the unmetabolized forms in edible tissues. Therefore, milk, meat, liver, heart, and eggs could be highly contaminated. Taken together, this information paves the way towards the development of a thorough monitoring plan for food and feed products to better guarantee animal and human safety and avoid economic losses. Considering the risks for both animal and human health, the European Commission defined strict regulations, together with MRLs. In this regard, it is important to mention the Commission Regulation (EU) 2023/915 [52] on maximum levels for certain contaminants in food, repealing Regulation (EC) No. 1881/2006.

**Figure 3:**
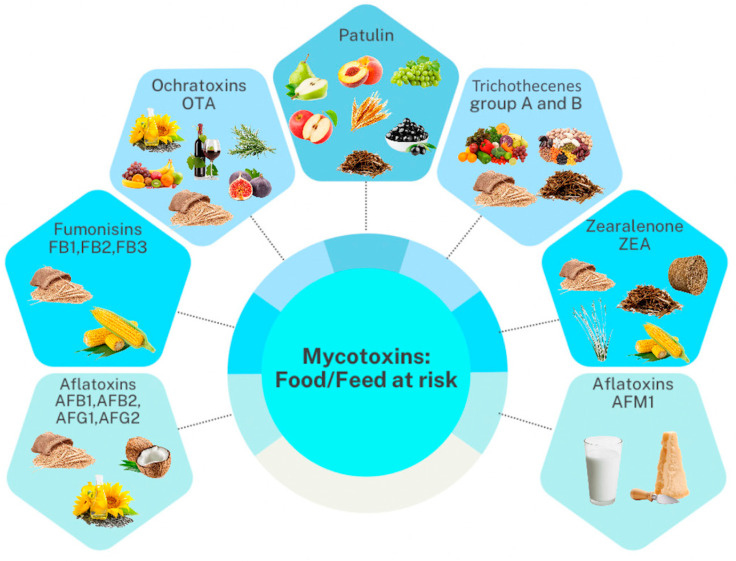
Relevant mycotoxin groups that contaminate different feed and food products.

Mycotoxin occurrence and levels in diverse feeds and foodstuffs have been recently reviewed by Ali et al. [53] and El-Sayed et al. [54]. We recommend these two references for a comprehensive understanding of mycotoxin contamination and the quantitative levels in food and feed matrices from different countries. Therefore, in this review, we summarized only the most frequent mycotoxins and their metabolites per main food and feed category (Figure 3).

Overall, mycotoxins occur in multiple feeds and food items at very different levels. Our literature search outlined that cereal grains and nuts are the most contaminated sources [53]. Also, we found that a high frequency and level of selected mycotoxins can occur regardless of the geographical origin of the product category [53]. Food products that were mainly contaminated were cereals (i.e., wheat, maize, oats), nuts (i.e., peanuts, hazelnuts), animal-derived products (e.g., milk, meat, eggs), mixed food products, and others (such as cocoa and soybean meal). The most widespread and consumed cereals have been widely examined in previous scientific works for mycotoxin contamination. A comprehensive three-year survey (2018–2020) by Khodaei et al. [55] on the global occurrence of mycotoxins in cereals revealed that the hazard of AFB1 in wheat, maize, and rice is serious, considering that these mycotoxins were higher than the EU limit in most of the reviewed cases. Overall, the high stability of mycotoxins during different phases of the production chains (i.e., production, distribution, storage, and processing) of cereals poses a high concern about the risks associated with the consumption of this food category [56]. Therefore, practical control measures combined with proper management strategies are mandatory to ensure consumer safety.

## 3. Challenges in the Analysis of Emerging and Hidden Mycotoxins

Because of climate change and the corresponding increase in fungi contamination in several matrices, the official control of mycotoxins and fungal metabolites in both animal feed and human food has recently gained even more importance than in previous years [57]. Today, more than 300 mycotoxins are known, but only a very small number are subjected to legal regulations. Interestingly, previous studies carried out on commercial animal feed samples from European and Mediterranean vs. Asian and Pacific areas revealed that more than half of the commercial samples were definitely contaminated by mycotoxins at a higher level than the corresponding legal limits [30]. It is important to consider that regulated mycotoxins can be structurally modified by fungi, plant, and animal metabolic reactions, thus resulting in potentially toxic metabolites not considered in legislation. More important is the case of emerging mycotoxins; these are a group of compounds not included in regulations and scarcely analytically determined. However, several recent literature reports have highlighted their toxicity and high co-occurrence [58]. Emerging mycotoxins (such as nivalenol, enniatins, beauvericin, diacetoxyscirpenol, fusaric acid, patulin, moniliformin, and sterigmatocystin) are widely present in cereals and other feed commodities worldwide [58]. It is also important to mention the challenges associated with the determination and quantification of so-called masked or hidden mycotoxins. These are biologically modified metabolites of mycotoxins with improved or reduced toxicity compared with parent mycotoxins and are difficult to detect through classical and/or targeted analytical methods [59]. These can be attached to carbohydrates or proteins, thus hampering their extraction from the matrix of interest through the available extraction protocols designed for different classes of toxins [59]. The modified and/or altered physicochemical properties are related to different chromatographic behaviors and are difficult to identify because of the lack of authentic standard compounds. Taken together, the information collected suggests that, in this way, potential underestimations of the total mycotoxin content of samples occur. A high number of scientific publications are available and focused on the potential hydrolysis of these modified or hidden forms of mycotoxins to re-generate toxic parent compounds. This event is common during mammalian digestion, thus determining severe toxicological concerns because these toxins are highly bioavailable in the digestive system [60,61]. Again, a concrete monitoring plan for these mycotoxin metabolites is still a main task for guaranteeing both food safety and human and animal health. In this complex scenario, ultra-high-performance liquid chromatography coupled with tandem mass spectrometry (UHPLC–MS/MS) can overcome the issues raised by modified mycotoxins and be used as a selective detection and quantification-based approach for several compounds in different feed, food, and/or biological matrices [62]. Considering that, nowadays, no legal regulations on chemically differentiated or modified mycotoxins in food and feed exist, more studies and scientific knowledge are needed to protect human and animal health.

As comprehensively reviewed by Okasha et al. [59], the occurrence or co-occurrence of masked mycotoxins is higher in food than in feed. The majority of the available research studies are based on the evaluation of modified forms of ZEN resulting from phase I and phase II biotransformations. However, although the biochemical pathways dealing with biotransformation are known [63], very few studies are available on other modified forms, such as acetyl DON derivatives, hydrolyzed FBs, and phase I metabolites T2 and NIV3G. In particular, the correlation between in vitro data and in vivo situations necessitates further investigation [63]. It seems that one of the major weapons available now against the few and/or inconsistent data reported on masked or hidden mycotoxins (mainly in terms of their toxicity) could be represented by the recent advancements in analytical methods. In this regard, the ability of high-resolution mass spectrometry tools to simultaneously detect multiple mycotoxins (both in their original and masked forms) makes it possible to overcome the previously mentioned limitations [62]. On the other side, the utilization of these high-throughput analytical methods poses limitations in terms of costs, and, sometimes, data are difficult to compare because of the absence of standardized protocols (mainly when considering sample collection and preparation) [59]. Therefore, this literature overview suggests and invites researchers working in this field to dedicate a great deal of attention to both free and masked mycotoxins in terms of developing new targeted and untargeted screening methods able to provide a beneficial snapshot of the contamination levels of a food or feed item or the potential toxicity associated with the presence of potentially toxic metabolites and/or hidden forms.

## 4. Transition from Targeted Analysis to Targeted/Untargeted Multiscreening-Based HRMS Approaches

In the last few years, research on mycotoxins has moved quickly towards the utilization of LC-MSMS and LC-HRMS approaches to determine multiple mycotoxins. Therefore, so-called multi-screening methods (for both qualitative and quantitative purposes) have started to be rapidly developed [62]. One of the major advantages of using HRMS over targeted MS/MS techniques is represented by the possibility to perform both untargeted and retrospective data analysis, with the latter allowing the reconsideration of analytical results from stored data and already analyzed samples. In particular, these methods are based on the measurement of accurate MS and MS/MS spectra with a mass resolution lower than 5 ppm. These conditions usually allow the detection of several compounds, together with the possibility to perform a structural elucidation of unknowns [64]. All these aspects represent a valuable tool because of the lack of analytical standards for several known mycotoxins. Untargeted and retrospective data analyses are particularly useful when modified and/or hidden mycotoxins are targeted, especially if one wants to investigate combined toxic effects [65,66]. Therefore, the utilization of full-scan methods based on high-throughput platforms (exploiting resolving powers up to 100,000 FWHM), such as Time-of-Flight (TOF) and Orbitrap, has been explored as a robust and complementary alternative to triple–quadrupole-based methods [62]. Also, the use of LC or UHPLC coupled with different HRMS platforms is extremely advantageous because the high resolution of these instruments provides information about targeted compounds, non-targeted compounds, and novel compounds in a single analytical run [62,67]. Therefore, the main advantages arising from the utilization of HRMS in mycotoxin research can be exploited in the field of emerging and modified mycotoxins because of the lack of certain analytical standards (sometimes not commercially available), calibrants, and reference materials. Also, as previously mentioned, there is an urgent trend of collecting more data about the co-occurrence of different regulated and emerging mycotoxins in both food and feed matrices [1] in order to better define a risk assessment on the potential synergistic effects of different compounds in terms of toxicity. On the other hand, an important issue associated with LC-HRMS method development, validation, and routine utilization is represented by the matrix effect. This latter can alter the ionization efficiency of the selected analyte in the presence of coeluting compounds, thus affecting both accuracy and sensitivity. Overall, according to the scientific literature [68], there are several possibilities for evaluating matrix effects. One of them is represented by the post-extraction spike method comparison, where three sets of samples are considered, namely, a) a neat standard dissolved in a selected solvent, b) a post-extraction spiked sample, and c) a pre-extraction spiked sample to also calculate extraction efficiency.

Overall, the comprehensive information gathered by HRMS utilization is useful for developing and improving novel toxin-based libraries and databases [69] and for further exploiting retrospective analyses of full-scan data [70]. The availability of qualitative occurrence and co-occurrence data could be exploited to better define food quality controls, thus ensuring the food safety and health of consumers. However, as comprehensively reviewed by Righetti et al. [62], HRMS still presents some critical aspects to be faced for its routine application in mycotoxins and/or contaminants analyses, including (among others) the cost of instrumentation and analyses, the need to perform multivariate statistical analyses to better extrapolate the biological meaning, the utilization of software (sometimes not open-access), and the availability of a huge storage system. Another important point associated with the fast development of HRMS platforms and data management is associated with issues of isobaric co-eluting compounds and unknown compound identification; these points should always be considered and solved to allow for the effective use of HRMS for food safety purposes. Recently, the introduction and development of ion mobility-based mass spectrometry (IMS) allowed the addition of a third dimension of separation based on the size, shape, and charge of ions, thus implementing so-called levels of annotation of omics technologies [71,72]. Interestingly, a database for mycotoxins was previously established, containing more than 100 traveling wave IMS-derived CCS values [73]. Therefore, the actual and next frontier of multi-mycotoxin research seems represented by coupling IMS and UHPLC-HRMS platforms in order to improve the quality of annotated mass features. Also, besides the important issue of isobaric compounds (which can be resolved neither by MS nor by UHPLC), this strategy could enhance compound identification by adding structural information (i.e., size and shape) based on CCS measurements [62]. Some previous applications of LC–ESI–TWIMS–TOF–MS methods (including CCS-filtering in data processing) have been exploited for the analysis of mycotoxins in cereals [73] and other contaminants (such as pesticides) in different feed and food items [74].

**Figure 4:**
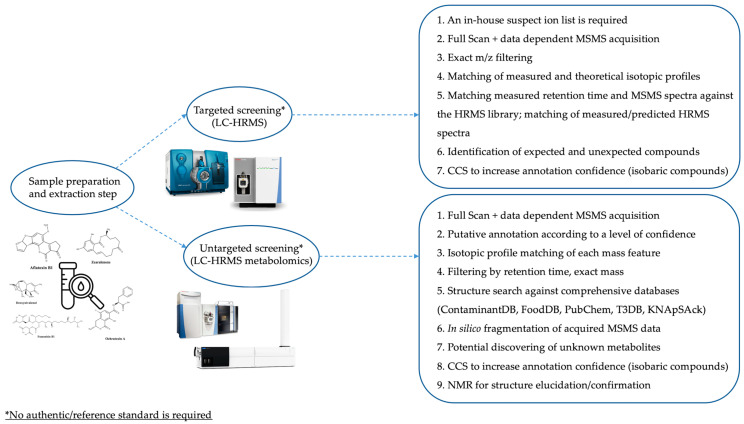
Workflows based on targeted and untargeted multiscreening for the detection of mycotoxins in different food and feed products. Adapted from [61].

A schematic overview of the main differences and identification-based workflows of targeted, untargeted, and multiscreening/retrospective methods is reported in Figure 4.

Some of the most recent applications dealing with the utilization of multi-LC-MSMS and multi-LC-HRMS for mycotoxin determination are reported in Table 2.

### 4.1. Applications of Multiscreening Methods for Biofluids

Lauwers et al. [75] developed an accurate multiscreening method for the quantification of mycotoxins in pig and chicken biofluids using LC-MSMS. The authors targeted mainly aflatoxins, ochratoxin A, and *Fusarium* mycotoxins, followed by emerging mycotoxins. Additionally, the same developed method was used under LC-HRMS conditions to qualitatively analyze phase I and II metabolites. Similarly, Tkaczyk and Jedzinak [76] proposed an LC-MS/MS method for the sensitive and selective analysis of 35 mycotoxins (as biomarkers of exposure) in pig urine samples. The authors revealed that DON, ZEN, and OTA were the main toxins detected in these samples.

### 4.2. Applications of Multiscreening Methods for Food Samples

Zhang et al. [77] developed a data-dependent acquisition-based HRMS (HPLC-Orbitrap) for tracing *Alternaria* mycotoxins and their sulfated metabolites in tomatoes, obtaining LOD and LOQ values of 0.009 and 0.030 μg/kg, respectively. A very interesting application of a multiscreening method for the simultaneous analysis of regulated, emerging, and modified mycotoxins was proposed by González-Jartín and co-authors [78] for milk; in particular, the authors developed a new method for the analysis of 40 mycotoxins in 31 cow milk samples using a QuEChERS extraction procedure to minimize the matrix effect. Overall, the authors demonstrated a high occurrence in these samples of *Fusarium* mycotoxins, namely, beauvericin and enniatins. BEA and ENNs are emerging mycotoxins frequently found in cereals and are mainly associated with adverse effects on the gastrointestinal tract. Similarly, looking at available data on milk samples, targeted analysis and retrospective screening of mycotoxins were carried out by Izzo et al. [79] using a UHPLC-HRMS approach. The authors analyzed 56 commercial milk samples from the Italian market, optimizing an alternative analytical tool for the simultaneous identification of 30 mycotoxins. Overall, no analyzed sample was contaminated by mycotoxins; however, the retrospective screening based on UHPLC-HRMS allowed for the tentative identification of other fungal and bacterial metabolites of interest. The potential for retrospective screening based on UHPLC-HRMS was also highlighted by Rocchetti et al. [80], who analyzed 45 bulk milk samples previously classified into different clusters according to the maize silage contamination profile. Generally, 14 compounds showed significant prediction ability when considering milk from contaminated and uncontaminated feeding systems, with antibiotic Y, bikaverin, and fumonisin B2 being the best predictive biomarkers. Among the discriminant metabolites, a high abundance of *Fusarium* mycotoxins was noticed, together with tetrapeptide tentoxin (an *Alternaria* toxin), α-zearalenol (a catabolite of zearalenone), mycophenolic acid, and apicidin. A multiscreening method was developed by Akinyemi and co-authors [81] to evaluate the mycotoxin contamination profile of raw milk from three different Nigerian animal species belonging to cow, goat, and camel. The authors analyzed 135 raw milk samples using an ultra-sensitive LC-MSMS method based on the determination of 36 different mycotoxins. The most abundant mycotoxins detected were aflatoxin P1, alternariol monomethyl ether, citrinin, dihydrocitrinone, enniatins, ochratoxin α, and sterigmatocystin, reported for the first time in animal milk. Interestingly, the most frequent mycotoxin detected in animal milk samples was beauvericin (87%), thus confirming the importance of emerging mycotoxins in the entire dairy food chain. In this scenario, Le et al. [82] developed a multiscreening method for mycotoxins in commercial cashew nut samples collected from Vietnam. The experimental conditions were based on QuEChERS extraction coupled with UPLC-MSMS to detect a total of 18 mycotoxins. The authors highlighted that the high heat resistance of mycotoxins represents a key factor facilitating their stability during conventional food processing. Also, cashews containing more additives had significantly higher FB1 concentrations, but not DON. Therefore, further studies are necessary to better correlate food processing conditions with mycotoxin formation in cashew nuts. Work by Li et al. [83] developed a method for the detection of mycotoxins in a complex matrix, such as vegetable oil. The comprehensive analysis of 20 commercial vegetable oils revealed that AFB1 and AFB2, followed by ZEN, were the most frequent mycotoxins observed. The authors stated that the method was simple and low-cost, thus promoting its wider applications for scanning mycotoxins in oil matrices. Recently, Sulyok et al. [84] reported the validation of an LC-MSMS method for the simultaneous quantification of more than 700 mycotoxins and other secondary fungal metabolites and plant toxins in pasta, biscuits, crackers, and muesli. Overall, application of the method to 147 samples from the EU market revealed the presence of enniatins and DON in the majority of samples.

### 4.3. Applications of Multiscreening Methods for Feed Samples

A comprehensive survey by Zhang et al. [85] was carried out on maize silage samples from the China region, known to be potentially contaminated with mycotoxins. The authors analyzed 200 maize silage samples collected in 2019 for regulated, masked, and some emerging mycotoxins. Moving to the main findings, the targeted multiscreening method revealed that DON and ZEN were the most frequent compounds in the analyzed samples, while the most commonly detected masked mycotoxin was 15-acetyldeoxynivalenol. Of interest, the results of this study pointed out the importance of beauvericin, which was detected in 99.5% of the samples analyzed with concentrations lower than 25 μg/kg. Also, the authors revealed that the accumulation of beauvericin was strongly correlated with DON and ZEA, thus indicating the urgent need to perform additional and comprehensive studies to better focus on masked and emerging mycotoxins. A previous application by González-Jartín et al. [86] allowed for the development of a multi-detection method for mycotoxins with modified QuEChERS extraction in feed. In particular, the authors outlined that maize and maize-based products were highly contaminated by toxins, although always below the legal limits. This might be of great concern since maize silage is one of the main components of milk cow feed in many regions of the world. Overall, the richness in protein and polysaccharides of maize cobs and leaves is reported to help fungi and other pathogens proliferate. Therefore, maize silage can be a dangerous source of AFB1, DON, and ZEN, with DON and ZEN being the most relevant ones in maize grains and whole maize. Another important and potential source of mycotoxins (because of their worldwide consumption) is represented by cashew nuts. A reliable, sensitive, and accurate multiple mycotoxin method was also developed by Nualkaw et al. [87] for the simultaneous determination of 17 mycotoxins in 300 feed samples (including swine, poultry, and dairy feeds) using stable isotope dilution (^13^C-ISTD) and UHPLC-MS/MS. The authors demonstrated that FUM, ZEN, AFB1, and DON highly contaminated the feed samples. Targeted analysis combined with retrospective screening allowed researchers to measure multiple mycotoxins in pet food; in particular, Castaldo et al. [88] used a comprehensive strategy based on a combination of quantitative methods for 28 mycotoxins and post-target screening for an additional 245 fungal and bacterial metabolites in dry pet food samples. Acetonitrile-based extraction was carried out, followed by the utilization of UHPLC-Q-Orbitrap (HRMS) as a detection method. The authors outlined the simultaneous occurrence of up to 16 toxins per sample.

## 5. Omics Technologies as a Valuable and Emerging Tool to Assess Mycotoxicity

As a recent research field, omics technologies, including metabolomics-based approaches, have been described as very useful in providing strong knowledge about mycotoxin metabolization in a host. Also, metabolomics is reported to provide a good level of knowledge of potential interactions with other biomolecules and targeted organs, allowing for the modeling of intraspecies variability and biochemical crosstalk [89]. In particular, omics-based approaches are valuable tools for the analysis of clinical samples, such as biofluids. This high-throughput technique can provide a comprehensive overview of the pathophysiological state mediated by mycotoxin exposure, thus helping to discover and define the most relevant exposure biomarkers related to adverse implications [90].

As previously stated, omics technologies based on HRMS allow researchers to consider all three potential forms of existing mycotoxins, namely, unmodified (parent) forms of mycotoxins biosynthesized by different fungi (e.g., AFs, OTA, ZEN, FBs, PAT, and DON), followed by matrix-bound mycotoxins such as those non-covalently complexed with proteins and polysaccharides, and chemically or biologically modified mycotoxins (masked or hidden) produced by fungi, bacteria, plants, or animals or derived from different food processing conditions [91]. Regarding biological samples, such as blood, it is therefore mandatory to consider in omics-based studies also those modified mycotoxins that have transformed back to their native forms in the digestive system and are potentially exerting similar toxic effects as the parent, thus contributing to final mycotoxin exposure.

Metabolomics is now considered a very robust and powerful technology to ensure the unbiased and overall determination of several metabolites (i.e., small metabolites with a mass range from 50 to 1500 Da). Therefore, this approach is finding wide application in different food matrices [89]. Targeted and untargeted metabolomics studies employ a combination of sample preparation, analytical tools, and computational analysis [92]. Among the most widespread analytical tools, it is possible to list NMR, followed by GC- and LC-HRMS. High-resolution mass analyzers allow for an extremely high putative identification level by, for example, using QTOF and Fourier transformation-based instruments (e.g., Orbitrap and ion cyclotron resonance). According to the available scientific literature, the most commonly analyzed biofluids for targeted and untargeted metabolomics profiling related to mycotoxin exposure are blood, serum, plasma, urine, and milk [88,89,90]. In this regard, targeted metabolomics is usually used for detecting different ranges of contamination by mycotoxins in foods, feeds, and agricultural products [93] as well as for checking biomarkers of exposure [94]. As recently reported by Owolabi et al. [90], untargeted metabolomics provides a robust and unbiased platform for mycotoxin research and a better evaluation of human and animal exposure, allowing for the identification of the best biomarkers to be monitored for guaranteeing food and feed safety.

An interesting application of an omics-based approach dealing with mycotoxins and biofluids was previously published by Rocchetti et al. [95], evaluating the metabolomics changes of milk due to mycotoxin-contaminated maize silage intake by dairy cows. The analysis was based on UHPLC coupled with Orbitrap-HRMS. Overall, 628 significant milk metabolites were strongly affected by the five levels of maize silage contamination. Interestingly, 78 metabolites showed a very high and significant prediction ability. Accordingly, sphingolipids, together with purine and pyrimidine-derived metabolites, were the most affected classes of compounds. Also, metabolomics revealed a pivotal role of oxidized glutathione in milk samples associated with the silage group contaminated by emerging *Aspergillus* toxins. Recently, the combination of stable isotopes with metabolomics allowed for the identification of newly identified biotransformation products in human cell models, such as ZEN-pyridoxine, DON-3-sulfate, DON-10-sulfonate 2, DON-10-glutathione, and DON-cysteine, thus improving the state-of-the-art and existing information about the biotransformation of ZEN and DON [96,97].

Besides LC-HRMS, a metabolomics approach can also be realized using different analytical techniques, such as NMR. Different samples, including rumen fluid, blood, and milk, collected from dairy cows were previously analyzed by Wang et al. [98] to obtain a comprehensive understanding of the metabolic changes resulting from AFB1 exposure. Particularly, the authors combined ^1^H-NMR spectroscopy with classical biochemical assays and outlined a significant impact of AFB1 exposure on amino acid metabolism (mainly phenylalanine) in all three biofluids. However, the authors concluded that mycotoxicity should be better assessed by using not only biomarkers of metabolomics but also some indicators of milk composition and production variables. Another interesting study was developed by Ogunade et al. [99] evaluating biomarkers of aflatoxin ingestion through a 1H-NMR-based metabolomics approach on dairy cows fed aflatoxin B1 with or without sequestering agents. The authors found that AFB1 greatly affected the plasma metabolomics of lactating Holstein dairy cows. In particular, a strong decrease in plasma amino acids (such as alanine, leucine, and arginine) and acetic acid was observed, while a corresponding increase in ethanol was detected. The latter was proposed as a good candidate biomarker of aflatoxin ingestion in dairy cows [99]. Another valuable example of a metabolomics-based approach dealing with mycotoxins and biofluids is represented by a study carried out by Gerdemann et al. [100] for the analysis of cellular alterations caused by 20 mycotoxins in HepG2 cells. Hydrophilic-based chromatography combined with targeted HRMS allowed for the selective and sensitive detection of more than 100 metabolites, easily associated with metabolic alterations caused by mycotoxins. In particular, the authors reported moniliformin and citrinin as the most significantly affected compounds for the citric acid cycle, also influencing glycolysis and energy metabolism. Penitrem A, ZEN, and T2 toxins mainly determined an imbalance of the urea cycle and amino acid homeostasis. The formation of ROS was associated with the presence of T2 toxins and gliotoxin, while OTA altered glycolysis. Finally, DNA synthesis was affected by several mycotoxins [100].

## 6. Conclusions and Future Perspectives

The presence of mycotoxins in food and feed is a severe problem affecting both quality and safety areas, as well as consumers’ health. The critical review of the available scientific literature here proposed suggests the need to switch from targeted to untargeted and/or retrospective screening analyses to better understand the exposure level and the potential toxicity of the very few studied mycotoxins and metabolites. The selection of samples, sampling, and extraction techniques are crucial for accurate and reliable results on mycotoxins. LC–HRMS is emerging, and it is today highly suitable for detecting multiple mycotoxins and modified mycotoxins in food and feed products, although the proper quantification and detection of masked/hidden mycotoxins in complex matrices is still one of the major obstacles. Additionally, the implementation of regulations for so-called emerging mycotoxins is still challenging. In this complex scenario, omics technologies are emerging as a valuable tool to comprehensively inspect the effect of mycotoxins on organisms by analyzing different biofluids (such as plasma and urine). Untargeted metabolomics allows researchers to check major changes in biochemical pathways together with the mode of action of different mycotoxins, although technical (due to sample preparation, the columns used, and their detection/analysis) and biological (due to different species targeted and/or different environmental/dietary factors) variability is still difficult to model in these studies. HRMS combined with an omics approach has the potential in the near future to be of great help to regulatory bodies to limit the presence of mycotoxins in food and feed; however, the lack of precise standard operating procedures makes the results of different laboratories difficult to compare. Therefore, this literature review supports the fast development of multi-omics approaches within a systems biology scenario; in this regard, genomics, transcriptomics, proteomics, and metabolomics have started to be integrated to create a more holistic comprehension of cells, organisms, and communities. The literature reviewed also shows that besides multi-omics evaluations of host–toxin interactions, another valuable strategy could be analyzing the multi-omics among host gut microbiomes–toxins in order to implement mitigation strategies. Finally, it is important to mention that machine learning approaches (such as convolutional neural networks) for both the detection and prediction of the presence of mycotoxins have seen a rise in recent years as an alternative to traditional detection methods (such as LC-HRMS), although there is an urgent need for reproducibility and transparency in machine learning research through open-access data and codes.

## Figures and Tables

**Table 1:** Legal limits for the most important mycotoxins in animal feeds, according to the European Commission recommendations. Adapted from [30].

Mycotoxin Group	Animal Species	Legal Limit (mg kg^−1^)
Aflatoxins (AFB1, AFB2, AFG1, AFG2)	Cows	0.005
	Other species	0.010
	Cow, goat, sheep, poultry	0.020
Type A-Trichothecenes (T-2 and HT-2 toxins)	All species	0.25
Type B-Trichothecenes (Deoxynivalenol, DON)	Swine	0.9
	Calves, lambs, and young goats	2.0
	Other species	5.0
Fumonisins (FB1, FB2, FB3)	Swine, equine, rabbits, and pet animals	5.0
	Poultry, calves, lambs, and young goats	20
	Adult ruminants and minks	50
Zearalenone (ZEN)	Piglets	0.1
	Fattening pigs	0.25
	Calves, cows, sheep, and goats	0.5
Ochratoxin A (OTA)	Swine	0.05
	Poultry	0.1

**Table 2:** Most interesting studies of the last five years dealing with the development of multiscreening methods to simultaneously detect different mycotoxins in different feed and food matrices.

Matrix	Multiscreening Method	Most Relevant Finding	Reference
Commercial grain products	UHPLC–ESI-Q/Orbitrap HRMS	Establishment of a customized accurate-mass database and mass spectral library for 63 mycotoxins. The method was highly sensitive, accurate, and high-throughput.	[69]
Biological matrices (plasma, urine, feces)	Targeted and untargeted LC-ESI-HRMS (Xevo^®^ TQ-S mass spectrometer)	Targeted quantification of regulated mycotoxins (AFs, OTA, and *Fusarium* mycotoxins) and emerging mycotoxins (Alternaria mycotoxins and ENNs)	[75]
Biological sample (urine)	HPLC-ESI-MSMS (QTRAP^®^ 6500 mass spectrometer)	Sensitive, selective, and simultaneous determination of 35 mycotoxins and metabolites like nivalenol, citrinin, dihydrocitrinone, fusarenon-X, altertoxin I, tentoxin, and hydrolyzed fumonisin B1.	[76]
Tomato samples	UHPLC-ESI-Q-Orbitrap	Detection of 24 sulfated metabolites, with two metabolites (AME-sulfated and AOH-sulfated) identified in *Alternaria* fungi-inoculated samples.	[77]
Raw milk	UHPLC-ESI-MSMS (Agilent 6460 triple quadrupole mass spectrometer)	Quantification of 40 mycotoxins in milk through QuEChERS extraction and UHPLC-MS/MS analysis. High occurrence (in low amounts) of beauvericin and enniatins in milk.	[78]
Commercial UHT milk (whole, semi-skim, and skim types)	UHPLC-ESI-Q-Exactive Orbitrap HRMS	Simultaneous analysis of 30 regulated and emerging mycotoxins through a sensitive, efficient, and quick method (chromatography run time of 8 min). No analyzed sample was contaminated with mycotoxins.	[79]
Bulk milk (from different dairy farms)	UHPLC-ESI-Q-Exactive Focus™ Orbitrap HRMS	*Fusarium* mycotoxins, together with tetrapeptide tentoxin, α-zearalenol, mycophenolic acid, and apicidin, were some of the most discriminant metabolites of the feeding system.	[80]
Raw milk (from different animal species)	Ultra-sensitive LC-MS/MS (Sciex QTrap^®^ 6500^+^ mass spectrometer)	Samples collected in Niger showed that at least one mycotoxin was detected in 97% of all samples, with BEA (87%) being the most frequent. AFM1 was absent in camel milk samples.	[81]
Commercial cashew nuts	UPLC-MS/MS system (TSQ Quantis triple quadrupole)	Optimization of an analytical method for the measurement of 18 mycotoxins in commercial samples from Vietnam. FB1 showed the highest variation according to the seasoning techniques.	[82]
Commercial vegetable oils	UHPLC-ESI-Q-Exactive Plus Orbitrap-MS	Aflatoxins (B1 and B2) and zearalenone were observed in 50% of the real samples analyzed, with a total of 10 real samples. The developed method was simple and low-cost, with great potential to screen mycotoxins in complex oil matrices.	[83]
Commercial grain products	UHPLC-QTrap 5500 MS/MS system	Simultaneous quantification of 730 mycotoxins and other secondary fungal metabolites and plant toxins. The enniatins and deoxynivalenol discriminated were found in the majority of the samples.	[84]
Commercial wheat flours	UHPLC-HRMS (Q-Exactive HF Orbitrap)	A screening method for detecting both parent and modified mycotoxins was developed. In particular, an in-house MS/MS database containing 82 mycotoxins divided into eight categories was constructed.	[85]
Maize and grass silage (from different dairy farms)	UHPLC–ESI-MS–IT–TOF combined with UHPLC-ESI-MS/MS	A high co-occurrence of *Fusarium* mycotoxins was found, with low contamination levels. DON and BEA were the most frequent compounds in silage (82%).	[86]
Swine, poultry, and dairy feeds	UHPLC-ESI- MS/MS (ExionLC™ AD system coupled with QTRAP 5500 tandem mass spectrometer)	FBs, ZEN, AFB1, and deoxynivalenol were the most prevalent mycotoxins in the samples analyzed.	[87]
Dry pet food	UHPLC-ESI-Q-Orbitrap HRMS	Comprehensive method combining quantification of 28 mycotoxins and post-target screening for another 245 fungal and bacterial metabolites. Emerging *Fusarium* mycotoxins were the most commonly detected mycotoxins.	[88]

## Data Availability

The original contributions presented in this study are included in the article, further inquiries can be directed to the corresponding author.

## Abbreviations

Aflatoxins (AFs), Aflatoxin B1 (AFB1), Aflatoxin B2 (AFB2), Aflatoxin G1 (AFG1), Aflatoxin G2 (AFG2), Aflatoxin M1 (AFM1), Fumonisins (FUMs), Fumonisins B (FBs), Fumonisin B1 (FB1), Fumonisin B2 (FB2), Fumonisin B3 (FB3), Trichothecenes (TCs), Deoxynivalenol (DON), Nivalenol (NIV), Zearalenone (ZEN), Ochratoxins (OTs), Ochratoxin A (OTA), Beauvercin (BEA), Enniatins (ENNs), Patulin (PAT), International Agency for Research on Cancer (IARC), European Food Safety Authority (EFSA), Reactive Oxygen Species (ROS), European Commission (EC), Maximum Residue Levels (MRLs), Liquid Chromatography (LC), Mass Spectrometry (MS/MS), Ultra-High-Performance Liquid Chromatography (UHPLC), Ultra-High-Performance Liquid Chromatography Coupled Tandem Mass Spectrometry (UHPLC-MS/MS), High-Resolution Mass Spectrometry (HRMS), Liquid Chromatography Coupled High-Resolution Mass Spectrometry (LC-HRMS), Liquid Chromatography Coupled Tandem Mass Spectrometry (LC-MS/MS), Time-of-Flight (TOF), Ion Mobility-Based Mass Spectrometry (IMS), Cross Collisional Sections (CCs), Traveling Wave Ion Mobility Spectrometry (TWIMS), Liquid Chromatography Electron Spry Ionization Coupled High-Resolution Mass Spectrometry (LC-ESI-HRMS), High-Performance Liquid Chromatography Electrospray Ionization Coupled Tandem Mass Spectrometry (HPLC-ESI-MS/MS), Ultra-High-Performance Liquid Chromatography Electrospray Ionization Coupled Tandem Mass Spectrometry (UHPLC-ESI-MS/MS), Ultra-High-Performance Liquid Chromatography Electrospray Ionization Coupled Tandem Mass Spectrometry (UHPLC-ESI-MS/MS), Ultra-High-Performance Liquid Chromatography Electrospray Ionization Coupled Quadrupole and High-Resolution Mass Spectrometry (UHPLC-ESI-Q-Orbitrap HRMS), Limit of Detection (LOD), Limit of Quantification (LOQ), Quick-Easy-Cheap-Effective-Rugged-Safe (QuEChERS), Quadrupole Time-of-Flight (QTOF), Nuclear Magnetic Resonance (NMR), Proton Nuclear Magnetic Resonance (1H-NMR).
